# Principles and Illustrations of Morbid Anatomy; Adapted to the Elements of M. Andral, and to the Cyclopædia of Practical Medicine, &c.

**Published:** 1834-07-01

**Authors:** 


*Principles and Illustrations of Morbid Anatomy; adapted to the Elements of* M. Andral, *and to the Cyclopædia of Practical Medicine, &c.* By J. Hope, m.d., f.r.s., Physician to the St. Marylebone Infirmary, &c.—London, 1833 and 4. 8vo. Parts i. to x. pp. 248 and lxxxiv; with numerous coloured Plates.

In our second Number we gave a short notice of the seventh part of this beautiful work; but, as ten parts are now lying before us, we trust that we shall gratify many of our readers by giving them a brief abstract of the contents of the whole book.

The first two parts comprise diseases of the lungs, such as acute peripneumony, abscess, pleurisy, phthisis, pulmonary apoplexy, emphysema, bronchitis, &c. In these, as throughout, the plates are remarkable for their fidelity to nature, as well as their pictorial beauty. The 46th figure gives a good representation of ulceration of the fauces and epiglottis, with vegetations extending into the larynx, together with hypertrophy of the mucous glands and papillæ. We quote the case annexed to it, because it is short and pithy enough to have obtained the author a prize in Sparta.

"Case. Syphilis treated with excess of mercury." (P. xiv.)

The third part contains an account of the diseases of the heart; and we give Dr. Hope great credit for not having yielded to the temptation of dilating too far on a subject, on which his long and zealous researches must have supplied him with a superabundance of materials. The 54th figure, representing the appearances on dissection in a case of acute pericarditis, deserves especial mention. The patient was "a man in La Charité, who had protracted peripneumony, in the last stage of which pericarditis supervened, and was suspected by Chomel, four days before death, by the pulse becoming very irregular; this being the only well-marked symptom. The pericardium contained eight ounces of turbid, flocculent, yellow serum." (P. xvii.)

The fourth and fifth parts are dedicated to the diseases of the liver, and form a good compendium of what is known, and what is conjectured, on its physiology and pathology.

The sixth, seventh, eighth, and ninth parts are devoted to diseases of the alimentary canal below the diaphragm.

The following observations, taken from the ninth part, are ingenious and interesting, though we would not pledge ourselves to the soundness of all the theories contained in them.

"*Seats of Hypertrophy.* There is no part of the canal below the diaphragm in which hypertrophy of the submucous cellular tissue has not been observed; but it occurs principally in the parts most liable to chronic inflammation of the mucous membrane, namely, the stomach, the rectum, the colon, and the end of the ileum. Hypertrophy of the muscular coat is found principally where that coat is naturally of the greatest thickness, namely, in the pylorus and pyloric third of the stomach, in the cardia, the rectum, and the colon.

"Contraction of the pylorus is occasionally attended with enormous dilatation of the stomach, which has been known to reach even to the ossa pubis. (*Andral, Clin. Med.*) Under these circumstances the walls are sometimes attenuated, and sometimes of natural thickness. The dilatation arises from distension of the organ, by accumulations of food which cannot pass through the pylorus. After the lapse of several days, the stomach, distended to the extreme, relieves itself by disgorging its contents; and hence, says Andral, arise those vomitings, so remarkable for their extreme copiousness, which supervene from time to time, (every eight or ten days, for instance,) in individuals labouring under contraction of the pylorus. When the contraction proceeds from carcinomatous tumours, the vomiting ceases when ulceration of the tumour leaves the orifice free; but the symptom recurs in proportion as the tumour is regenerated. It occasionally happens that similar dilatation of the stomach takes place when the pyloric orifice is enlarged; an anomaly which is explained by the thickened walls of the viscus having lost their tonic contractile power; whence they allow accumulations to take place, as in an inert bag. When the pylorus is much thickened, a tumour may sometimes be felt externally; and, when the whole stomach is diffusely thickened and contracted, without forming any particular tumour, an unusual resistance is, in some cases, perceptible over the whole epigastrium.

In the small intestines, hypertrophy of the submucous tissues is rare, and it is generally confined to a particular spot, of small extent. Sometimes the symptoms of stricture are produced by it, and increase from time to time to those of strangulation, of which the patient, after several relapses, usually dies. The intermittent nature of the symptoms of strangulation is ascribed by Andral to temporary tumefaction of the mucous membrane of the contracted portion; but it is also certain, that the mere circumstance of distension of the gut above the stricture will, in contractions of a certain form, tend to close the passage; as, for example, in the stricture, fig. 177. In these cases the gut above the stricture is sometimes permanently dilated to three or four times its natural capacity.

In the colon, hypertrophy is more common at its two ends than in the middle; for the simple reason, that the mucous membranes and follicles are more subject to disease at the two ends. The thickening is in general attended with contraction of the gut; but sometimes the reverse takes place, and the walls, having lost their contractility, become dilated into a cyst with thick and hard parietes, forming adhesions with the contiguous parts, and presenting externally the feel of an abdominal tumour. (See case 105, by Abercrombie, on the Abdominal Viscera.)

Stricture of the rectum, or of the colon, (fig. 177,) is in general nothing more than hypertrophy of the submucous tissue (*a*, *a*,) succeeding to irritation and thickening of the mucous membrane itself.

"Condylomata around the anus are formed by hypertrophy of the submucous cellular tissue, covered with the thickened and more or less injected mucous membrane within the gut, and by the cuticle exterior to it. Fig. 179 represents a good specimen of the disease. The pale part, *a*, is a segment (about one fourth) of the sphincter ani: all exterior to it is a triangular portion of one side of the nates. The alteration sometimes consists of distinct rounded tumours, like hæmorrhoids, with necks narrower than their heads, as seen at *b*, *c*, *d;* in other cases there is only a diffuse, granulated thickening around the anus, as at *e*, where the diseased skin is a third of an inch thick; but most commonly this thickening and round tumours coexist, as in the present figure. The surface of the disease is highly vascular. During life the portion represented was universally red; but, after death, the blood gravitated away from the parts *a*, *c*, leaving them lead coloured, and stagnated in the large tumors, *b*, and other dependent parts, rendering them of an intense violet hue. On cutting them, blood oozed freely on pressure. When the thickened cuticle was peeled off, the surface had the granular appearance of a common wart, and the tumors grated like scirrhus, when divided with the scalpel. Sooner or later suppuration and ulceration take place, and the disease then assumes appearances often designated by the term *cancer*. In connexion with external condylomata, Andral once found similar globular bodies, of livid red colour, studding the whole internal surface of the rectum.

"Hypertrophy of the intestinal canal occurs most frequently between the ages of thirty-five and sixty-five, and is very rare between puberty and thirty-five. It is not uncommon in children between the ages of four and twelve, when of unhealthy constitutions, and subject to chronic diarrhœa.

"There is reason to believe that the blood-vessels and nerves of the intestinal canal may become hypertrophous (*Path. Anal.* ii. 86.) Hypertrophy of the lymphatic apparatus is a familiar affection; this being the nature of the enlargements of the mesenteric glands following protracted inflammation of the intestinal mucous membrane, as in figs. 154 and 155." (P. 213-216.)

The tenth part contains a short chapter on External Cancer, and the commencement of the account of diseases of the Uterine System.

Our imperfect list of the subjects treated of by the author, together with our long quotation, may give our readers some notion of the variety and extent of the topics discussed in this work. These Illustrations deserve great praise, yet there is a very serious defect in them: they contain too much of Andral, and Laennec, and Cruveilhier, and too little of Dr. Hope. The author will reply, that he gave us fair notice of his intentions on the title-page; but we must still rejoin, that we would rather that he had written a book substantive than a book adjective. However, had the author committed a hundred errors greater than this, the coloured drawings have merit enough to redeem them.

